# The *Pseudomonas aeruginosa* PAO1 metallo flavoprotein d-2-hydroxyglutarate dehydrogenase requires Zn^2+^ for substrate orientation and activation

**DOI:** 10.1016/j.jbc.2023.103008

**Published:** 2023-02-11

**Authors:** Joanna A. Quaye, Giovanni Gadda

**Affiliations:** 1 Department of Chemistry, Georgia State University, Atlanta, Georgia, USA; 2 Department of Biology, Georgia State University, Atlanta, Georgia, USA; 3 Department of The Center for Diagnostics and Therapeutics, Georgia State University, Atlanta, Georgia, USA

**Keywords:** *Pseudomonas aeruginosa* D-2-hydroxyglutarate dehydrogenase, D-2-hydroxyglutarate, d-malate, reductive-half reaction, Zn^2+^, metallo flavoprotein, flavin, catalytic mechanism

## Abstract

*Pseudomonas aeruginosa* PAO1 d-2-hydroxyglutarate (D2HG) dehydrogenase (*Pa*D2HGDH) oxidizes D2HG to 2-ketoglutarate during the vital l-serine biosynthesis and is a potential therapeutic target against *P. aeruginosa. Pa*D2HGDH, which oxidizes d-malate as an alternative substrate, has been demonstrated to be a metallo flavoprotein that requires Zn^2+^ for activity. However, the role of Zn^2+^ in the enzyme has not been elucidated, making it difficult to rationalize why nature employs both a redox center and a metal ion for catalysis in *Pa*D2HGDH and other metallo flavoenzymes. In this study, recombinant His-tagged *Pa*D2HGDH was purified to high levels in the presence of Zn^2+^ or Co^2+^ to investigate the metal's role in catalysis. We found that the flavin reduction step was reversible and partially rate limiting for the enzyme’s turnover at pH 7.4 with either D2HG or d-malate with similar rate constants for both substrates, irrespective of whether Zn^2+^ or Co^2+^ was bound to the enzyme. The steady-state pL profiles of the *k*_cat_ and *k*_cat_/*K*_*m*_ values with d-malate demonstrate that Zn^2+^ mediates the activation of water coordinated to the metal. Our data are consistent with a dual role for the metal, which orients the hydroxy acid substrate in the enzyme’s active site and rapidly deprotonates the substrate to yield an alkoxide species for hydride transfer to the flavin. Thus, we propose a catalytic mechanism for *Pa*D2HGDH oxidation that establishes Zn^2+^ as a cofactor required for substrate orientation and activation during enzymatic turnover.

Many higher organisms suffer from diseases caused by *Pseudomonas aeruginosa* (1, 2, 3, 4, 5, 6, 7, 8, 9, 10, 11, 12, 13, 14). Treatment of *P. aeruginosa* infections requires the use of antibiotics; however, the bacterium has developed multidrug resistance against the existing antibiotics (1, 2, 3, 6, 15), making treatments challenging with fatal nosocomial infections in humans (16, 17). Because of multidrug resistance, there is a need for new strategies to combat *P. aeruginosa* infections. *P. aeruginosa* d-2-hydroxyglutarate dehydrogenase (*Pa*D2HGDH) has been identified as a potential therapeutic target because it plays a vital role in *P. aeruginosa*'s life cycle (18). Upon D2HGDH gene knockout from the genome of the bacterium, *P. aeruginosa*’s growth is inhibited because of the lack of a compensatory activity that mitigates the effects of the D2HGDH gene removal on the bacterium (19, 20).

*Pa*D2HGDH has been reported as a Zn^2+^ and FAD-dependent enzyme that catalyzes the conversion of D2HG or d-malate to 2-ketoglutarate or oxaloacetate, respectively (18, 21). The Zn^2+^ requirement as an essential cofactor classifies *Pa*D2HGDH as a member of the poorly understood class of metallo flavoenzymes (21). *Pa*D2HGDH is also active with Co^2+^, Ni^2+^, Mn^2+^, and Cd^2+^ as alternative metal cofactors (21). Likewise, increased enzyme activity has been observed for several flavin-dependent enzymes following the addition of exogenous amounts of divalent metals such as Zn^2+^, Ni^2+^, Co^2+^, Mn^2+^, and Cd^2+^ (22, 23, 24, 25, 26, 27). The catalytic roles of metals in metalloenzymes (24, 28, 29, 30, 31, 32, 33, 34, 35) and flavins in flavoenzymes (23, 36, 37, 38, 39, 40, 41, 42, 43, 44, 45, 46, 47, 48) are established. However, the role of metals in metallo flavoenzymes is less explored (19, 21, 27, 49, 50, 51, 52, 53, 54, 55), making it difficult to rationalize why nature employs both a redox center and a metal ion for catalysis in enzymes such as *Pa*D2HGDH. Therefore, there is a need for in-depth studies on the mechanistic role of Zn^2+^ in *Pa*D2HGDH.

In metallo flavoenzymes, metals may be involved in substrate binding or flavin reduction (50, 56, 57, 58). It is essential to consider that the specific role of a metal in a protein would depend on the intrinsic property of the metal coupled with the enzyme's chemistry and active-site properties. Zinc, unlike other transition metals, is known to exist as a redox-inert Zn (II) cation (Zn^2+^) with an electronic configuration of [Ar]3d10, which makes it diamagnetic (59). In proteins, Zn^2+^ is known to be coordinated by soft base ligands like cysteine and histidine or hard base ligands like aspartate and glutamate. Thus, Zn^2+^ adopts different coordination numbers and binding geometries in biological systems. Because of its redox-inert state and versatile coordination geometries, Zn^2+^ has been shown to be abundant with broad functions in biology (59, 60, 61, 62, 63, 64, 65, 66, 67, 68) and is found in several enzymes including the well-characterized alcohol dehydrogenase, carboxypeptidase A, and thermolysin (60, 69, 70, 71, 72, 73, 74).

Zn^2+^ mainly functions as a catalyst, a cocatalyst, a structural ion, or an interface ion in proteins (59). Catalytic Zn^2+^ is known to be coordinated by three protein ligands and a water molecule, which is activated to a hydroxide ion because of the polarizing effect of the Zn^2+^ ion (63). Cocatalytic Zn^2+^ forms a catalytic unit with one or more Zn^2+^ or other transition metals, with histidine and glutamate, or aspartate, as the main ligands and water as a bridge in some cases (63, 75). Structural Zn^2+^ is generally coordinated by four protein ligands, mainly cysteine, with no water molecules (63, 75). Interface Zn^2+^ binds the surface between two protein subunits or interacting proteins with either catalytic or structural coordination (63). Based on previous reports on metalloproteins and the available data on *Pa*D2HGDH, the enzyme-bound Zn^2+^ is expected to directly impact enzyme catalysis, considering that the metalloapoenzyme is inactive (21). However, the mechanism of action of Zn^2+^, and for that matter, many other metals in metallo flavoproteins, are not fully understood.

In this study, His-tagged *Pa*D2HGDH from *P. aeruginosa* PAO1 has been recombinantly expressed, purified to high levels with Zn^2+^, and investigated for its kinetic properties using steady-state and rapid reaction kinetics employing pH, solvent kinetic isotope, and kinetic solvent viscosity effects. A catalytic mechanism has been proposed for substrate oxidation by the Zn^2+^-bound *Pa*D2HGDH using data from the reductive-half reaction, pL profiles, and solvent kinetic isotope effects on the *k*_cat_ and *k*_cat_/*K*_*m*_ values with d-malate as a substrate. This study also reports on the activity of *Pa*D2HGDH with an alternative metal, Co^2+^, consistent with previous data that the enzyme can utilize other divalent metals for catalysis (21). The data demonstrate that the metal orients the hydroxy acid substrate in the enzyme’s active site and rapidly deprotonates the substrate to yield an alkoxide species for hydride transfer to the flavin.

## Results

### Steady-state kinetics of E-Zn^2+^ and E-Co^2+^

To characterize the Zn^2+^-loaded *Pa*D2HGDH (E-Zn^2+^), the steady-state kinetic mechanism of the enzyme was determined with varying concentrations of D2HG or d-malate as a substrate and phenazine methosulfate (PMS) as an electron acceptor. Initial reaction rates were monitored using a Clark-type oxygen electrode monitoring the PMS-driven oxygen consumption reporting on enzyme turnover in 25 mM NaPO_4_, pH 7.4, at 25 °C. The best fit of the data was obtained using an equation describing a steady-state kinetic model with an irreversible kinetic step between the substrate and electron acceptor binding to the enzyme, yielding the steady-state kinetic parameters shown in Table 1.

**Table 1:** Steady-state kinetics of E-Zn^2+^^a^

Kinetic parameters	D2HG	d-Malate
*k*_cat_, s^−1^	26 ± 1	52 ± 2
*K*_*m* (substrate)_, mM	0.08 ± 0.01	6.0 ± 0.6
*K*_*m* (PMS)_, mM	0.07 ± 0.01	0.030 ± 0.003
*k*_cat_/*K*_*m* (substrate)_, M^−1^ s^−1^	330,000 ± 43,000	9000 ± 900
*k*_cat_/*K*_*m* (PMS)_, M^−1^ s^−1^	370,000 ± 55,000	1,700,000 ± 190,000

a Steady-state enzyme activity was measured at varying concentrations of D2HG, d-malate, and PMS. All assays were carried out in 25 mM NaPO_4_, pH 7.4, at 25 °C.

To characterize the Co^2+^-loaded *Pa*D2HGDH (E-Co^2+^), the steady-state kinetic mechanism of E-Co^2+^ was determined with varying concentrations of D2HG or d-malate and PMS as substrates, as described previously for E-Zn^2+^, yielding similar results and the steady-state kinetic parameters shown in Table 2.

**Table 2:** Steady-state kinetics of E-Co^2+^^a^

Kinetic parameters	D2HG	d-Malate
*k*_cat_, s^−1^	71 ± 4	46 ± 2
*K*_*m* (substrate)_, mM	0.11 ± 0.01	4.3 ± 0.3
*K*_*m* (PMS)_, mM	0.36 ± 0.03	0.10 ± 0.01
*k*_cat_/*K*_*m* (substrate)_, M^−1^ s^−1^	650,000 ± 65,000	11,000 ± 900
*k*_cat_/*K*_*m* (PMS)_, M^−1^ s^−1^	200,000 ± 20,000	460,000 ± 46,000

a Steady-state enzyme activity was measured at varying concentrations of D2HG, d-malate, and PMS. All assays were carried out in 25 mM NaPO_4_, pH 7.4, at 25 °C.

### Rapid reaction kinetics of E-Zn^2+^ and E-Co^2+^

**Figure 1:**
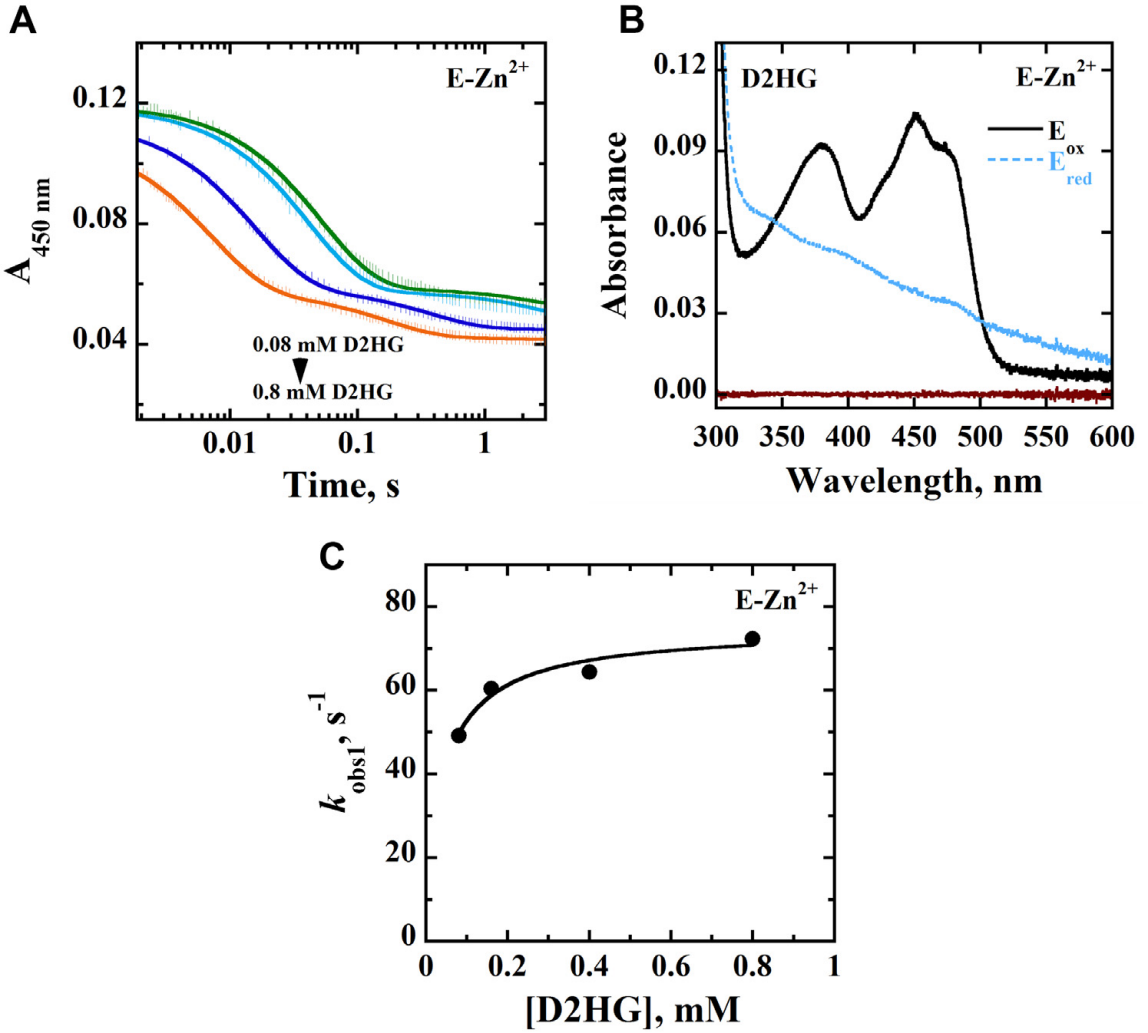
**Anaerobic reduction of E-Zn**^**2+**^**with D2HG as substrate.***A,* stopped-flow traces of *Pa*D2HGDH-Zn^2+^ at 450 nm with varying concentrations of D2HG (0.08–0.8 mM), fit to Equation 1. Note the log time scale. *B,* absorption spectra of *Pa*D2HGDH-Zn^2+^ showing the fully oxidized flavin before reduction (*black trace*), fully reduced flavin after reduction with 0.8 mM D2HG (*blue trace*), and reaction buffer (*brown trace*). *C,* concentration dependence of D2HG on *k*_obs1_ with *Pa*D2HGDH-Zn^2+^ fit to Equation 2. Assay was carried out in 25 mM NaPO_4_, pH 7.4 using an SF-61DX2 Hi-Tech KinetAsyst high-performance stopped-flow spectrophotometer thermostated at 25 °C and equipped with a photomultiplier detector under anaerobic conditions. The instrumental dead time is 2.2 ms. D2HG, d-2-hydroxyglutarate; *Pa*D2HGDH, d-2-hydroxyglutarate dehydrogenase from *Pseudomonas aeruginosa*.

**Figure 2:**
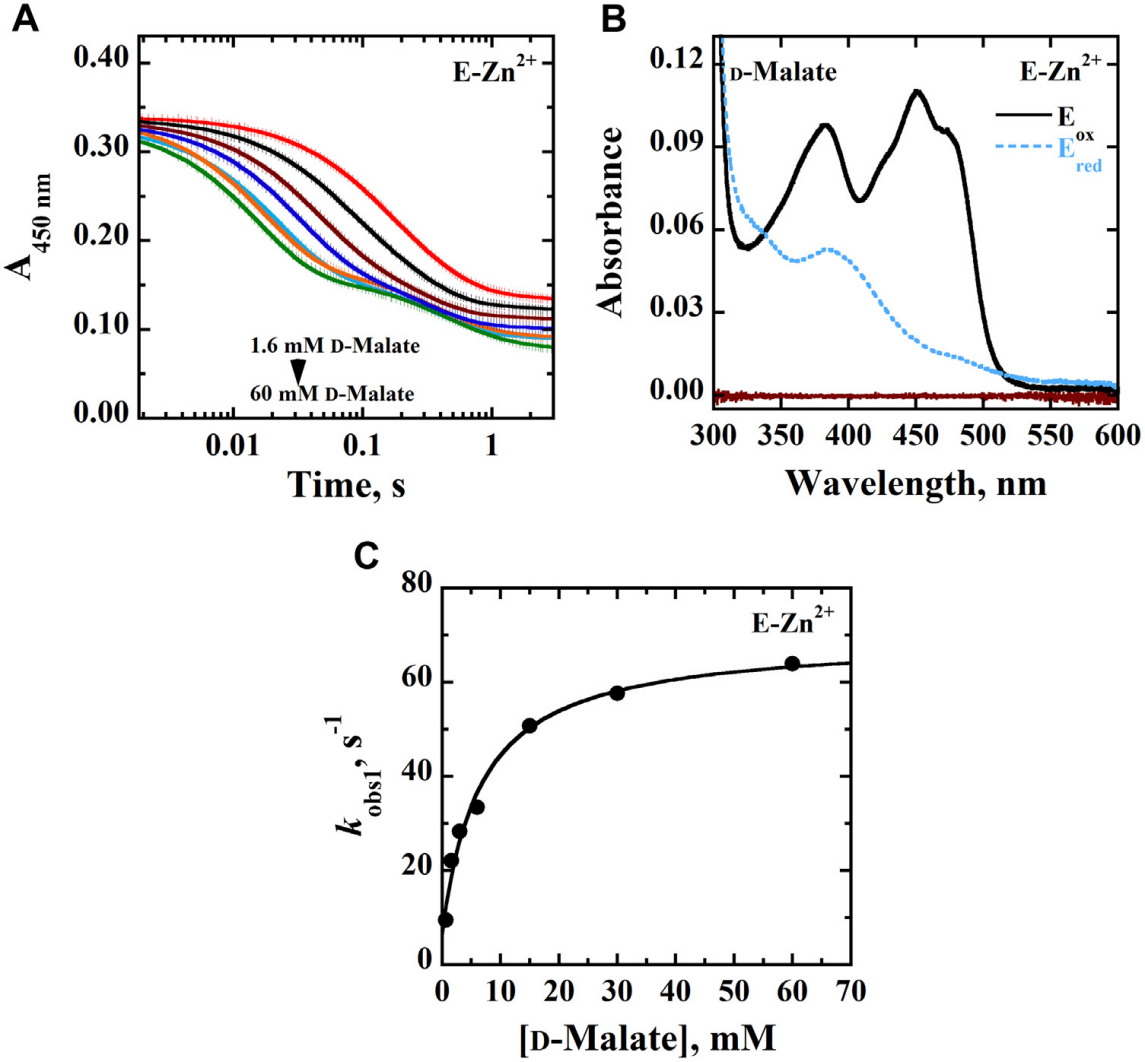
**Anaerobic reduction of E-Zn**^**2+**^**with****d****-malate as substrate.***A,* stopped-flow traces of *Pa*D2HGDH-Zn^2+^ at 450 nm with varying concentrations of d-malate (1.6–80 mM) fit to Equation 1. Note the log time scale. *B,* absorption spectra of *Pa*D2HGDH-Zn^2+^ showing the fully oxidized flavin before reduction (*black trace*), fully reduced flavin after reduction with 15 mM of d-malate (*blue trace*), and reaction buffer (*brown trace*). *C,* concentration dependence of d-malate on *k*_obs1_ with *Pa*D2HGDH-Zn^2+^ fit to Equation 2. Assay was carried out in 25 mM NaPO_4_, pH 7.4 using an SF-61DX2 Hi-Tech KinetAsyst high-performance stopped-flow spectrophotometer thermostated at 25 °C and equipped with a photomultiplier detector under anaerobic conditions. The instrumental dead time is 2.2 ms. *Pa*D2HGDH, d-2-hydroxyglutarate dehydrogenase from *Pseudomonas aeruginosa*.

The time-resolved anaerobic reduction of E-Zn^2+^ was investigated using a stopped-flow spectrophotometer by monitoring the loss of absorbance of the oxidized flavin at 450 nm upon mixing the enzyme with D2HG or d-malate at pH 7.4 and 25 °C. With both substrates, a full reduction of the enzyme-bound flavin was observed (Figs. 1 and 2). Pseudo–first order conditions with ∼9 μM enzyme and 80 to 800 μM D2HG or 0.6 to 60 mM d-malate were maintained, and the resulting stopped-flow traces were fit to a double exponential process with Equation 1 in which the fast phase accounted for ∼90% of the total absorbance change. The observed rate constants for the fast phase *k*_obs1_ were hyperbolically dependent on D2HG or d-malate concentration (Figs. 1 and 2), allowing for the determination of the limiting rate constant for flavin reduction *k*_red_ (Table 3). With d-malate, the best fit was obtained by fitting the kinetic data for the observed rate of flavin reduction with Equation 2 yielding a *y*-intercept consistent with a reversible step of flavin reduction *k*_rev_ (Fig. 2 and Table 3). The apparent equilibrium constant for the dissociation of the substrate from the Michaelis complex *K*_*d*_ could be determined for d-malate but not D2HG (Table 3), since the *K*_*d*_ value for D2HG was below the tested range of substrate concentrations. The observed rate constants for the slow phase *k*_obs2_, which accounted for ∼10% of the total absorbance change at 450 nm, were independent of the D2HG or d-malate concentrations with values between 1.1 and 3.1 s^−1^.

**Table 3:** Rapid reaction kinetics of E-Zn^2+^^a^

Kinetic parameters	D2HG	d-Malate
*k*_red_, s^−1^	74 ± 3	68 ± 2
*k*_rev_, s^−1^	ND^b^	4 ± 2
*K*_*d* (substrate)_, mM	<0.08	8 ± 1
*k*_red_/*K*_*d* (substrate)_, M^−1^ s^−1^	>930,000	8500 ± 1000

Abbreviation: ND, not determined.

a The anaerobic rapid enzyme reaction was measured at varying concentrations of D2HG or d-malate. All assays were carried out in 25 mM NaPO_4_, pH 7.4, at 25 °C under anerobic conditions.

b The *K*_*d*_ value was too low to be determined, thus the *k*_4_ value could not be measured.

**Figure 3:**
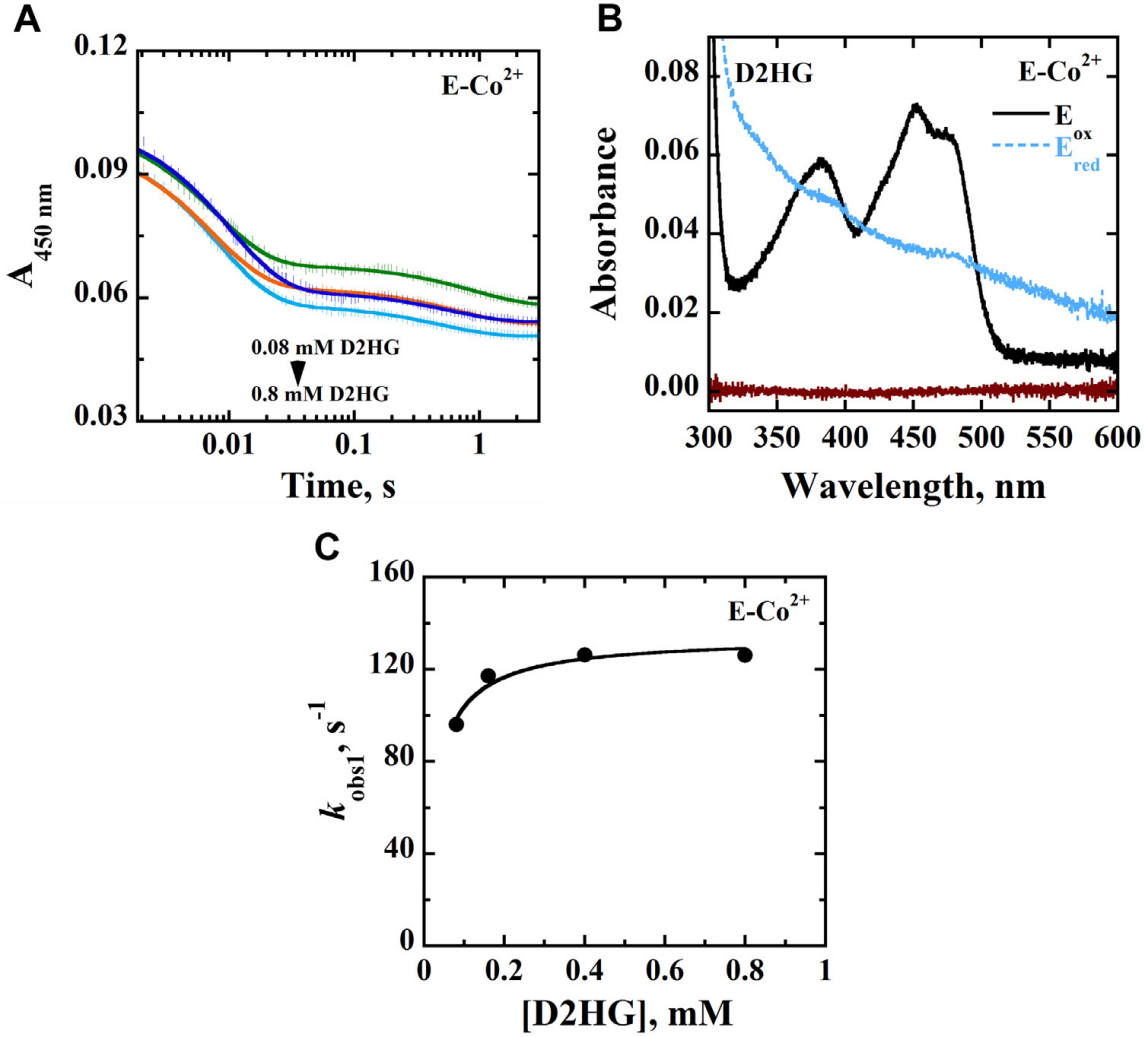
**Anaerobic reduction of E-Co**^**2+**^**with D2HG as substrate.***A,* stopped-flow traces of *Pa*D2HGDH-Co^2+^ at 450 nm with varying concentrations of D2HG (0.08–0.8 mM), fit to Equation 1. Note the log time scale. *B,* absorption spectra of *Pa*D2HGDH-Co^2+^ showing the fully oxidized flavin before reduction (*black trace*), fully reduced flavin after reduction with 0.8 mM D2HG (*blue trace*), and reaction buffer (*brown trace*). *C,* concentration dependence of D2HG on *k*_obs1_ with *Pa*D2HGDH-Co^2+^ fit to Equation 2. Assay was carried out in 25 mM NaPO_4_, pH 7.4 using an SF-61DX2 Hi-Tech KinetAsyst high-performance stopped-flow spectrophotometer thermostated at 25 °C and equipped with a photomultiplier detector under anaerobic conditions. The instrumental dead time is 2.2 ms. D2HG, d-2-hydroxyglutarate; *Pa*D2HGDH, d-2-hydroxyglutarate dehydrogenase from *Pseudomonas aeruginosa*.

**Figure 4:**
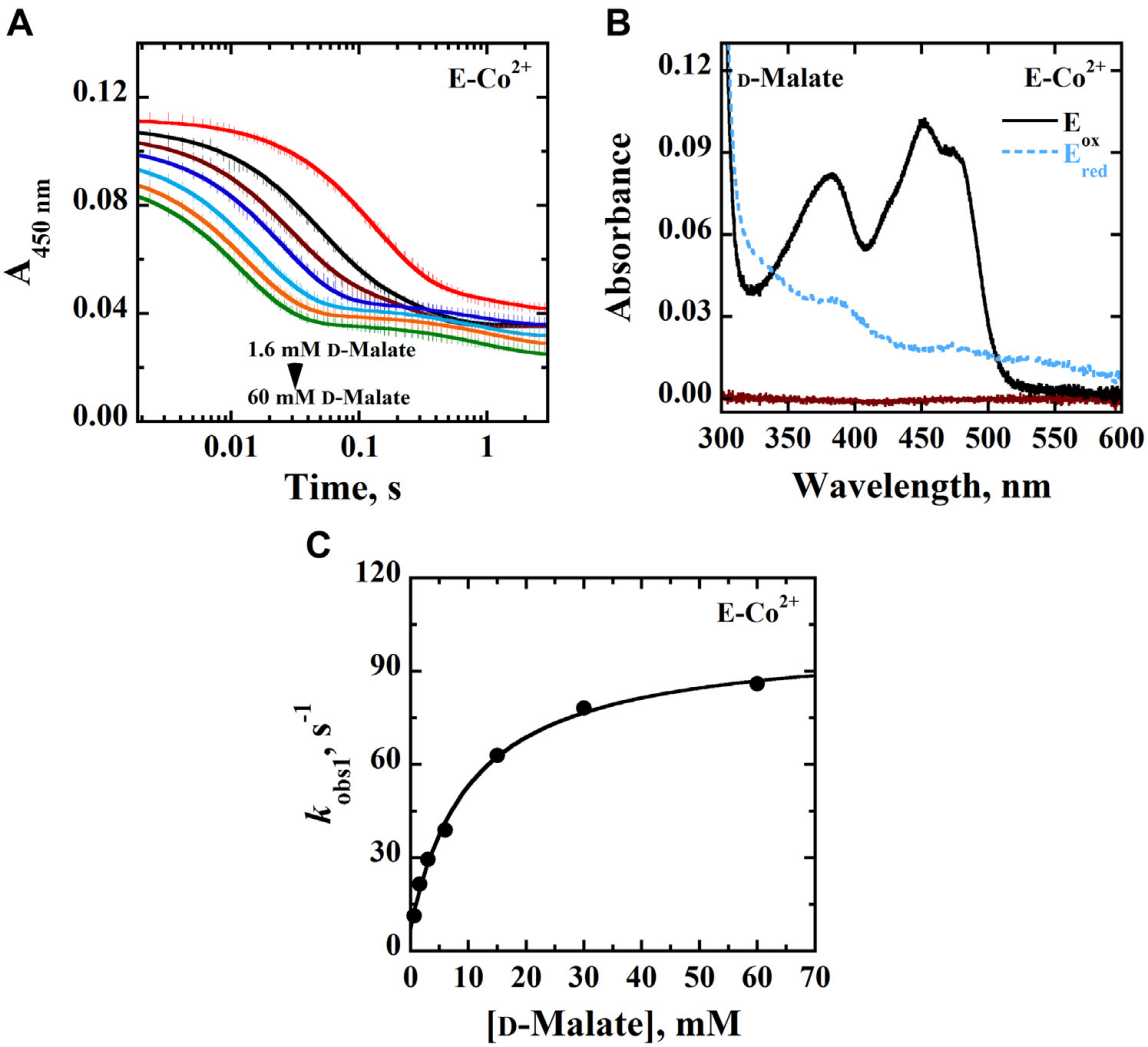
**Anaerobic reduction of E-Co**^**2+**^**with****d****-malate as substrate.***A*, stopped-flow traces of *Pa*D2HGDH-Co^2+^ at 450 nm with varying concentrations of d-malate (1.6–80 mM) fit to Equation 1. Note the log time scale. *B,* absorption spectra of *Pa*D2HGDH-Co^2+^ showing the fully oxidized flavin before reduction (*black trace*), fully reduced flavin after reduction with 15 mM of d-malate (*blue trace*), and reaction buffer (*brown trace*). *C,* concentration dependence of d-malate on *k*_obs1_ with *Pa*D2HGDH-Co^2+^ fit to Equation 2. Assay was carried out in 25 mM NaPO_4_, pH 7.4 using an SF-61DX2 Hi-Tech KinetAsyst high-performance stopped-flow spectrophotometer thermostated at 25 °C and equipped with a photomultiplier detector under anaerobic conditions. The instrumental dead time is 2.2 ms. *Pa*D2HGDH, d-2-hydroxyglutarate dehydrogenase from *Pseudomonas aeruginosa*.

When the time-resolved anaerobic reduction with D2HG or d-malate was investigated for E-Co^2+^, kinetic patterns and absorption spectra similar to those for E-Zn^2+^ were observed (Figs. 3 and 4), yielding the *k*_red_, *k*_rev_, and *K*_*d*_ values shown in Table 4.

**Table 4:** Rapid reaction kinetics of E-Co^2+^^a^

Kinetic parameters	D2HG	d-Malate
*k*_red_, s^−1^	130 ± 10	93 ± 3
*k*_rev_, s^−1^	ND^b^	9 ± 1
*K*_*d* (substrate)_, mM	<0.04	10 ± 1
*k*_red_/*K*_*d* (substrate)_, M^−1^ s^−1^	>3,250,000	9300 ± 1000

Abbreviation: ND, not determined.

a The anaerobic rapid enzyme reaction was measured at varying concentrations of D2HG or d-malate. All assays were carried out in 25 mM NaPO_4_, pH 7.4, at 25 °C under anerobic conditions.

b The *K*_*d*_ value was too low to be determined, thus the *k*_4_ value could not be measured.

### Kinetic solvent viscosity effects on E-Zn^2+^

**Figure 5:**
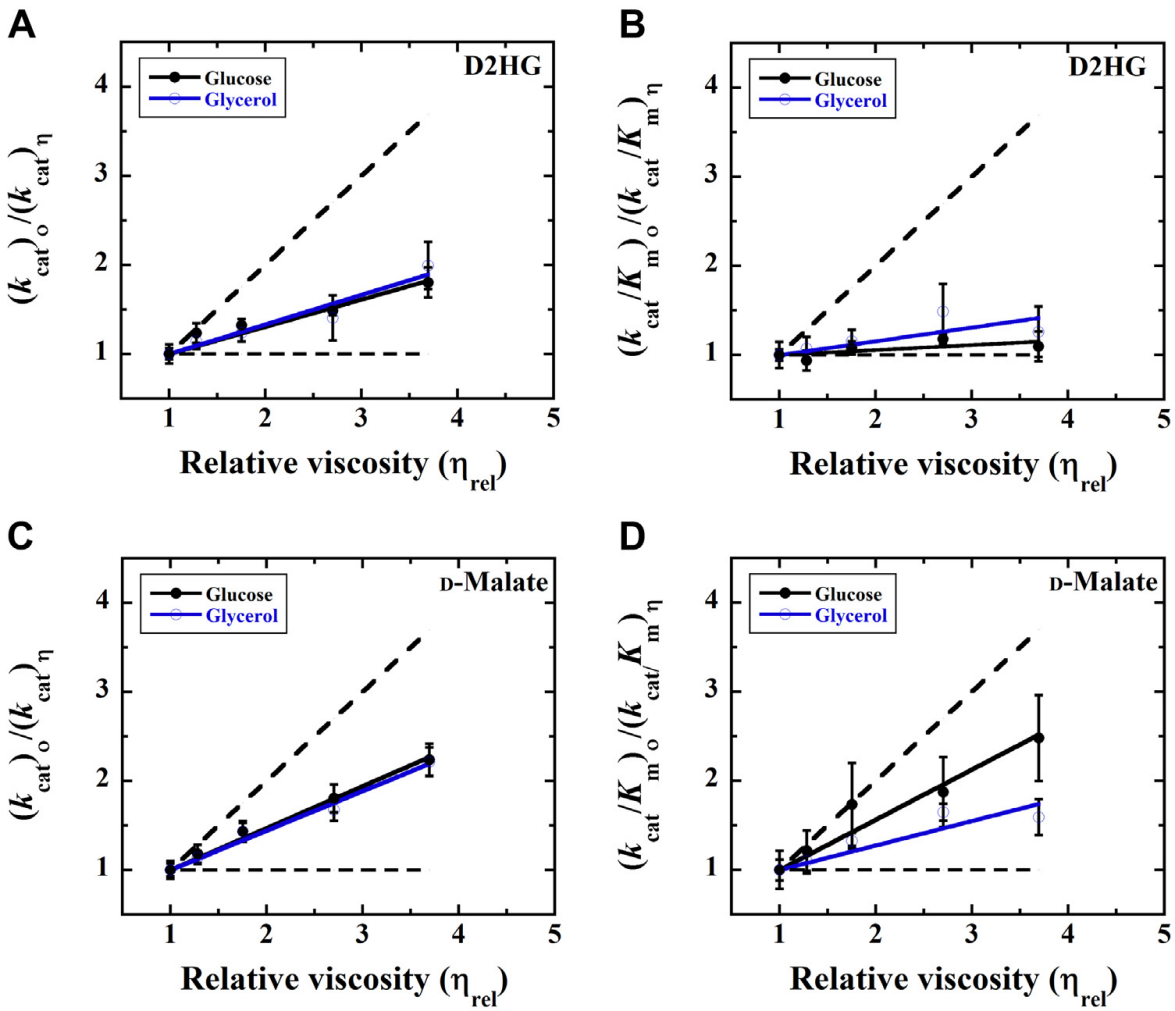
**Effects of solvent viscosity on steady-state kinetic parameters of E-Zn**^**2+**^**.***A,* Viscosity effects on the *k*_cat_ parameter with the physiological substrate D2HG. *B,* Viscosity effects on the *k*_cat_/*K*_m_ parameter with the physiological substrate D2HG. *C,* Viscosity effects on the *k*_cat_ parameter with the alternate substrate d-malate. *D,* Viscosity effects on the *k*_cat_/*K*_m_ parameter with the alternate substrate d-malate. The *dashed line* with a slope of 1 and slope of 0 describe a case in which the reaction is diffusion controlled and not affected by diffusion, respectively. *Pa*D2HGDH activity assays were carried out of in 25 mM NaPO_4_, pH 7.4 using a Clark-type oxygen electrode in 25 mM NaPO_4_, 1 mM PMS, pH 7.4 thermostated at 25 °C, containing varying amounts of glycerol: *blue* (0–40%, m/m, η_rel_ = 1.0–3.5 cP) and glucose: *black* as a viscogen (0–34%, m/m, η_rel_ = 1.0–3.6 cP). The *solid lines* report a fit of the data to Equation 3. The slopes of the viscosity effects on the normalized *k*_cat_ and *k*_cat_/*K*_*m*_ values with D2HG and d-malate are reported in Table 5. D2HG, d-2-hydroxyglutarate; *Pa*D2HGDH, d-2-hydroxyglutarate dehydrogenase from *Pseudomonas aeruginosa*; PMS, phenazine methosulfate.

Kinetic solvent viscosity effects were determined to investigate the rate-limiting steps for the overall turnover of E-Zn^2+^ with D2HG or d-malate and to gain insights into the kinetic steps involved in the substrate capture of E-Zn^2+^. The *k*_cat_ and *k*_cat_/*K*_*m*_ values with D2HG or d-malate were determined at varying concentrations of glycerol or glucose as an added viscosigen at pH 7.4 and 25 °C (76). As shown in Figure 5, the plots of the normalized *k*_cat_ and *k*_cat_/*K*_*m*_ values as a function of the relative solvent viscosity yielded straight lines with positive slopes for all viscosigens tested (Table 5). These data establish that the overall turnover and the reductive half-reaction of the enzyme are partially limited by diffusional processes.

**Table 5:** Kinetic solvent viscosity effects on the steady-state kinetic parameters of E-Zn^2+^^a^

Kinetic parameters		D2HG	d-malate
*k*_cat_	Glucose	0.31 ± 0.03	0.47 ± 0.01
	Glycerol	0.33 ± 0.03	0.44 ± 0.02
*k*_cat_/*K*_*m*_	Glucose	0.06 ± 0.02	0.56 ± 0.05
	Glycerol	0.15 ± 0.04	0.27 ± 0.04

a *Pa*D2HGDH activity assays were carried out in 25 mM NaPO_4_, pH 7.4 using a Clark-type oxygen electrode in 25 mM NaPO_4_, 1 mM PMS, pH 7.4 thermostated at 25 °C, containing varying amounts of glycerol (0–40%, m/m, η_rel_ = 1.0–3.5 cP) or glucose as a viscogen (0–34%, m/m, η_rel_ = 1.0–3.6 cP). The slopes from the viscosity effects were extrapolated after fitting the data to Equation 3.

### pL profile of E-Zn^2+^

**Figure 6:**
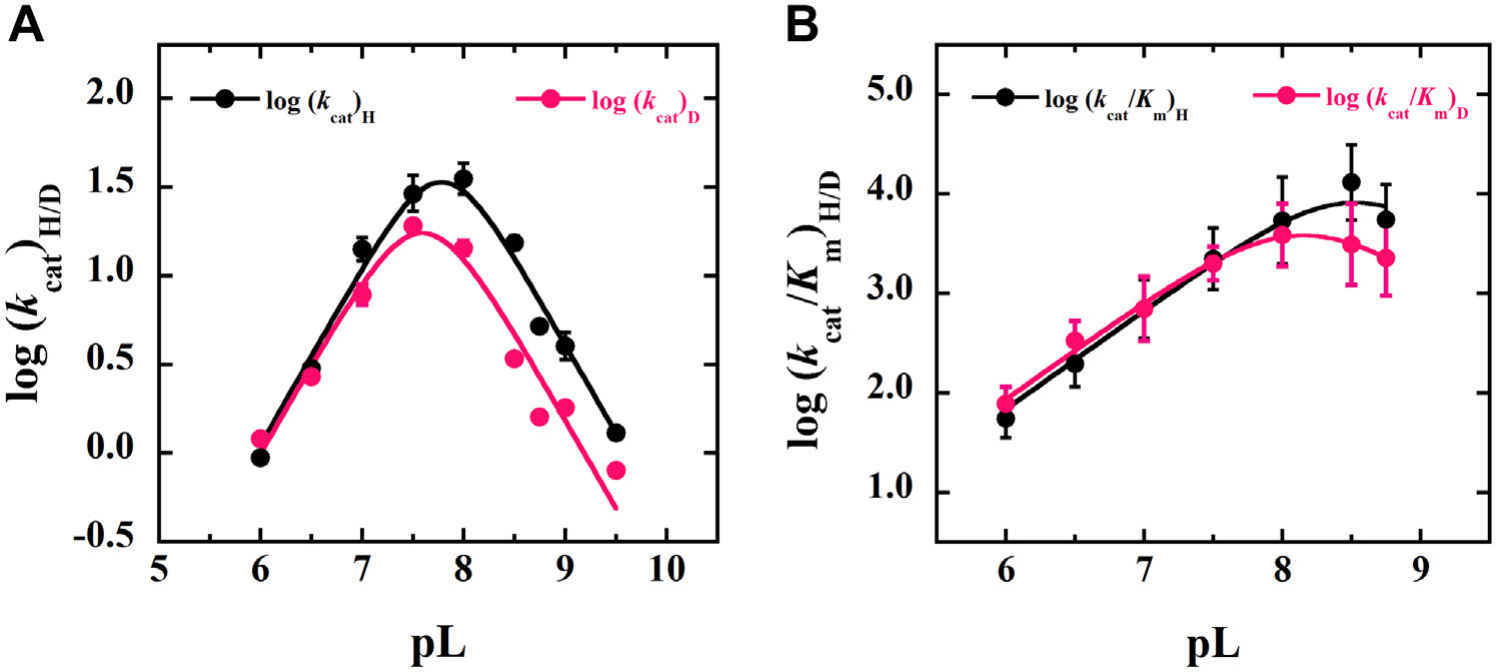
**pL dependence of the solvent kinetic isotope effects on the steady-state kinetic parameters of *Pa*D2HGDH with****d****-malate as substrate.***A,* pH dependences of the *k*_cat_ in H_2_O (*black*) and D_2_O (*pink*). *B,* pH dependences of the *k*_cat_/*K*_*m*_ in H_2_O (*black*) and D_2_O (*pink*). Values were determined by using the apparent steady-state approach with varying concentration of d-malate as substrate for *Pa*D2HGDH and fixed PMS concentration at 2 mM between pH 6.0 and 9.5, at 25 °C. The curves were obtained by fitting the kinetic data to Equation 4. *Pa*D2HGDH, d-2-hydroxyglutarate dehydrogenase from *Pseudomonas aeruginosa*; PMS, phenazine methosulfate.

The effects of pL on the steady-state kinetic parameters of E-Zn^2+^ with d-malate and PMS were determined in the pL range of 6.0 to 9.5 to investigate the p*K*_a_ values of groups participating in catalysis and proton exchange during catalysis. The steady-state kinetics followed a normal hyperbolic pattern, allowing for the estimation of the *k*_cat_ and *k*_cat_/*K*_*m*_ values at most pL values. The log *k*_cat_ values in H_2_O and deuterium oxide (D_2_O) were fit to Equation 4 and yielded bell-shaped curves (Fig. 6) for unprotonated and protonated groups that limit overall enzyme turnover. The apparent p*K*_a_ values in H_2_O and D_2_O were between 7.0 and 8.0, which were too close to be resolved. The log *k*_cat_/*K*_*m*_ values also appeared bell-shaped with apparent p*K*_a_ values of ∼8.0 for an unprotonated group in both H_2_O and D_2_O, but with insufficient data points to resolve the high pL limbs (Fig. 6).

## Discussion

**Figure 7:**
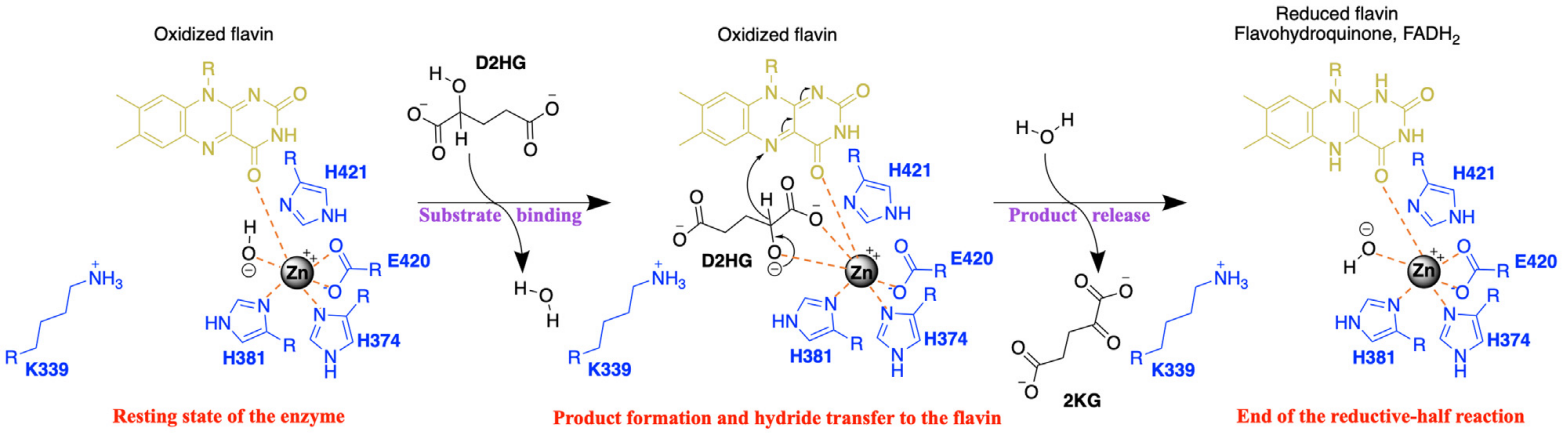
**Proposed catalytic scheme of the *Pa*D2HGDH-Zn**^**2+**^**reductive-half reaction.***Pa*D2HGDH, *P. aeruginosa*d-2-hydroxyglutarate dehydrogenase.

In the present study, metal-bound *P. aeruginosa* PAO1 D2HGDH has been characterized in its kinetic properties using steady-state and rapid-reaction kinetics and investigated for the effects of solvent viscosity, pH, and deuterated solvent on its kinetic parameters. The results presented are consistent with the minimal catalytic mechanism of Figure 7, which also considers recently reported structural data (18, 77). In the resting state, the active site Zn^2+^ coordinates to H^374^, H^381^, E^420^, the flavin O^4^ atom, and a hydroxide ion. Once the α-hydroxy acid substrate binds, the polarizing effect of Zn^2+^ results in the rapid deprotonation of the substrate C^2^-OH group yielding a substrate alkoxide that coordinates Zn^2+^ in a bidentate fashion together with the C^1^ carboxylate. The loss of the proton from the substrate hydroxyl triggers a hydride transfer to the flavin, resulting in the α-keto acid formation and flavin reduction. After product release, a water molecule binds to Zn^2+^ and is polarized to generate a hydroxide ion. Evidence supporting the proposed catalytic mechanism is discussed later.

### Zn^2+^ orients the hydroxy acid substrate in the active site for hydride transfer

**Figure 8:**
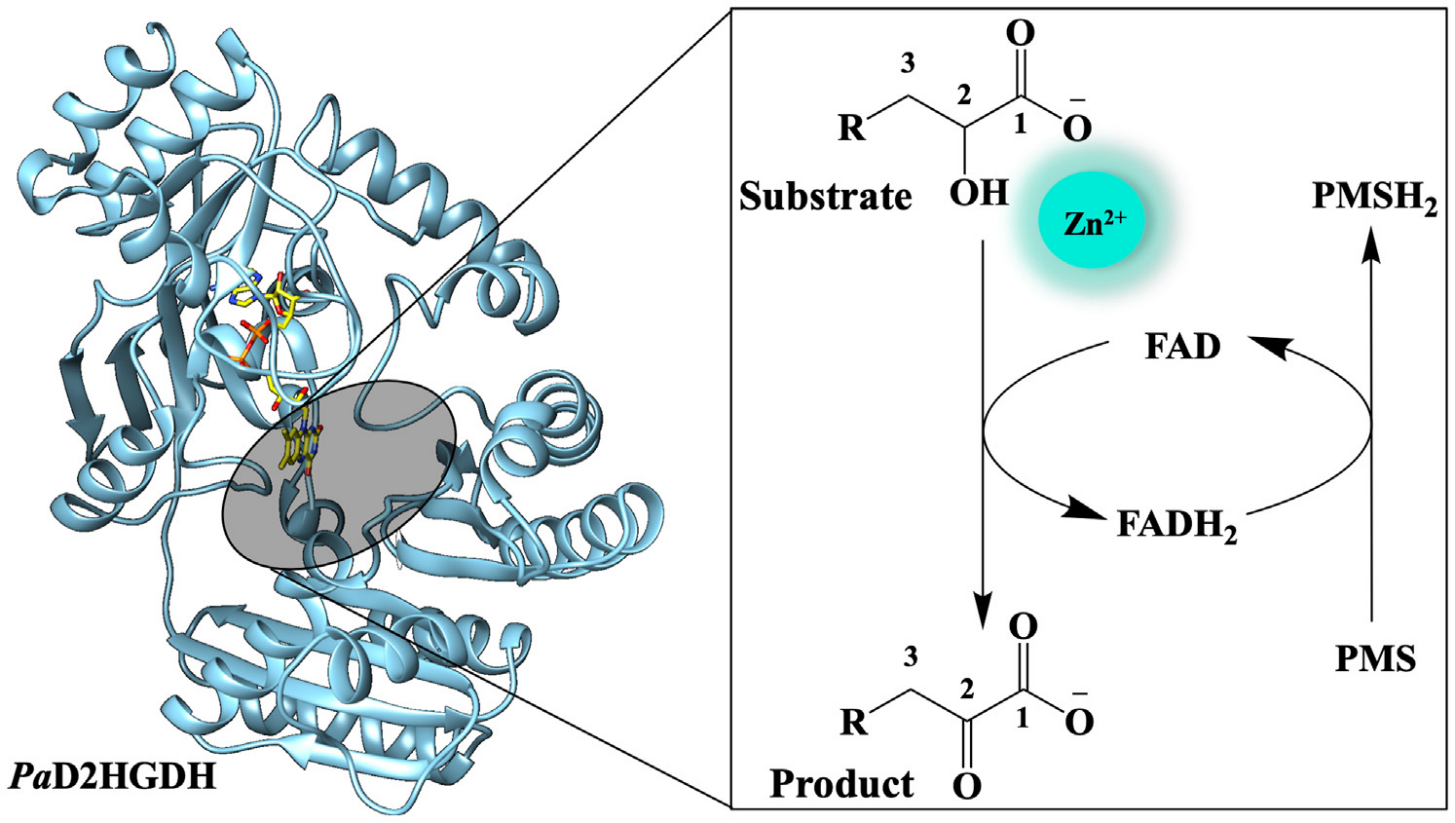
**General reaction scheme of *Pa*D2HGDH showing metal coordination with substrate.** R is CH_2_COO^−^ for d-2-hydroxyglutarate or COO^−^ for d-malate. *Pa*D2HGDH, *P. aeruginosa*d-2-hydroxyglutarate dehydrogenase.

**Figure 9:**
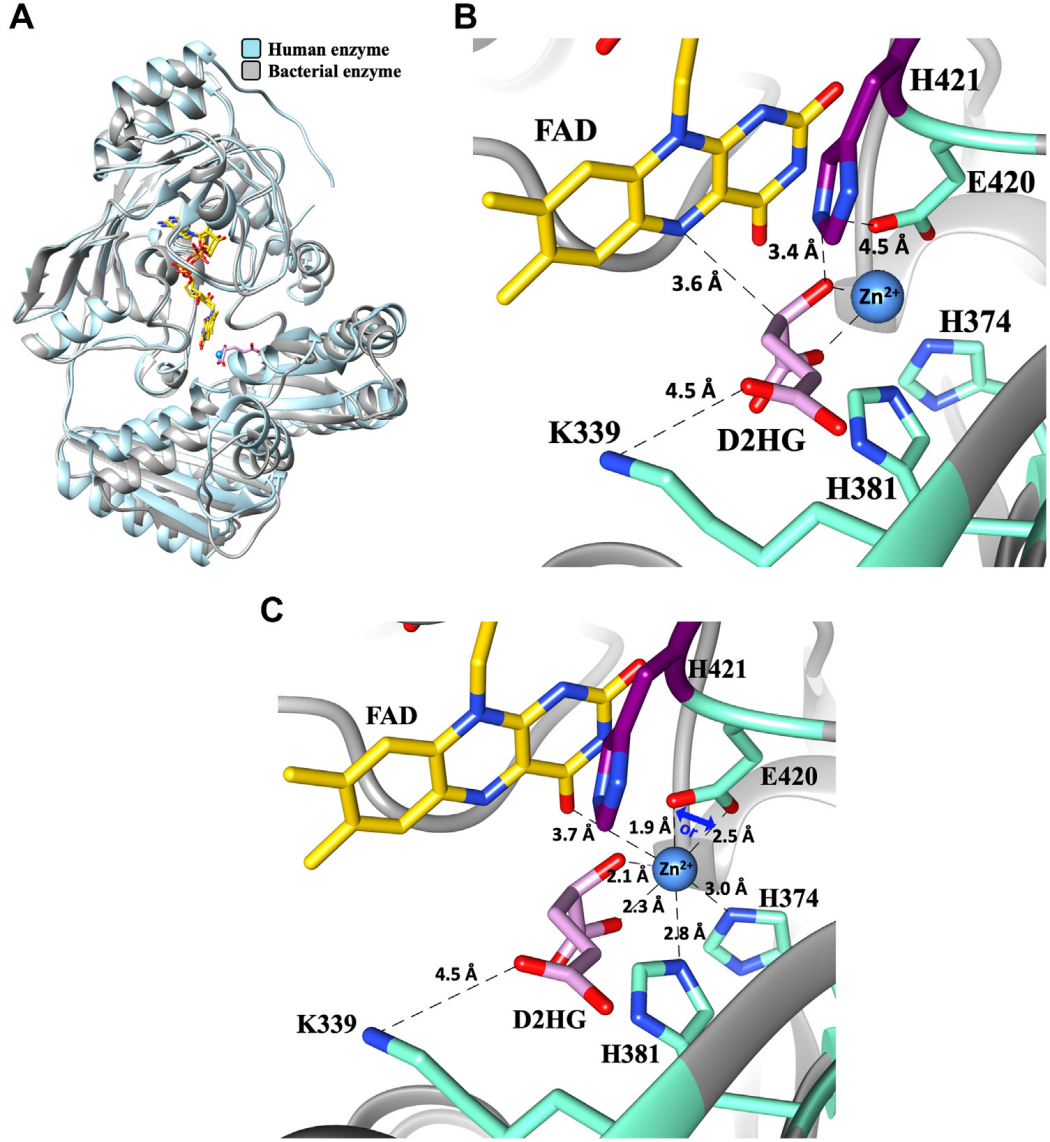
**Proposed binding interactions for Zn**^**2+**^**and substrate in the active site of *Pa*D2HGDH.***A,* an overlay of the homology model of *Pa*D2HGDH (18) (built with SWISS-MODEL using a putative dehydrogenase from *Rhodopseudomonas palustris* (Protein Data Bank code: 3PM9) and human D2HGDH (Protein Data Bank code: 6LPP) (77). *B,* active site of *Pa*D2HGDH homology model showing distances of the substrate C_2_ atom from the flavin reactive center (N^5^) and the proposed catalytic base, H^421^ (*dark purple*). *C,* active site of *Pa*D2HGDH homology model showing proposed ligands for Zn^2+^ coordination. Zn^2+^ binds with either of the E^420^ carboxylate oxygens and binds with only one at a time. *Pa*D2HGDH, d-2-hydroxyglutarate dehydrogenase from *Pseudomonas aeruginosa*.

Evidence to support this conclusion comes from the reductive-half reaction of the enzyme at pH 7.4 and 25 °C. The rate constant *k*_red_ for hydride transfer to the flavin was ∼70 s^−1^ when D2HG or d-malate was used to reduce E-Zn^2+^ (Table 3). The simplest rationale that explains the same rate constant for hydride transfer from four and five carbon-length substrates is a binding geometry involving bidentate metal coordination with the substrate C^1^ carboxylate and C^2^ hydroxyl oxygen atoms, as illustrated in Figure 8. Such a metal interaction would ensure that the substrate C^2^ atom donating the hydride ion to the flavin has the same orientation and distance from the flavin N^5^ atom that receives the hydride, irrespective of the substrate chain length. A bidentate metal coordination of the substrate would yield similar results when Zn^2+^ is replaced by other metals. Indeed, when the rapid-reaction kinetics of E-Co^2+^ was investigated, similar *k*_red_ values of ∼100 s^−1^ were obtained with D2HG and d-malate (Table 4). Structural data consistent with the mechanistic conclusion of bidentate metal coordination comes from a recent report on human D2HGDH, which shares fully conserved active-site residues and an overall fold with a *Pa*D2HGDH homology model previously built using a putative dehydrogenase from *Rhodopseudomonas palustris* as a template (Fig. 9) (18). The crystal structure of the human enzyme in a complex with D2HG or d-malate revealed Zn^2+^ coordination of both substrates in a bidentate interaction with the C^1^ carboxylate and C^2^ hydroxyl oxygens, positioning the C^2^ atom of D2HG or d-malate ∼3.1 Å from the flavin N^5^ atom (77).

### Zn^2+^ activates the hydroxy acid substrate for hydride transfer

This conclusion is supported by the pL profile of the *k*_cat_ values for E-Zn^2+^ with d-malate as substrate in H_2_O or D_2_O (Fig. 6), showing a bell-shaped pattern. At low pL values, the Zn^2+^-mediated ionization of the substrate C2-OH group that triggers the hydride transfer to the enzyme-bound flavin is unfavored, yielding low *k*_cat_ values. At high pL values with *Pa*D2HGDH, the hydride transfer to the enzyme-bound flavin is inhibited despite substrate ionization being favored. A possible rationale that explains the decreasing *k*_cat_ value with pH increasing above 8.0 is the ionization of the flavin N3 atom yielding an anionic flavin that prevents the hydride transfer from the substrate. The alternative rationale proposed for other metalloenzymes devoid of a flavin that the negatively sloped limb is due to an enzyme group donating a proton in the catalytic step cannot be ruled out for *Pa*D2HGDH based on the available data (78, 79, 80). Nevertheless, the catalytic mechanism of *Pa*D2HGDH is different from the established mechanism for other flavin-dependent alcohol-oxidizing enzymes that utilize a histidine to activate the hydroxy acid substrate for facile hydride transfer to the enzyme-bound flavin^1^ (81, 82, 83, 84, 85, 86).

### Hydride transfer is reversible in *Pa*D2HGDH

**Figure 10:**
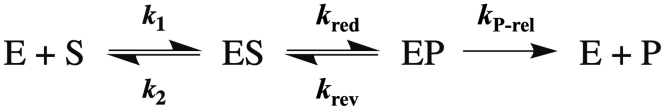
**Anaerobic reductive-half reaction pathway of *Pa*D2HGDH-Zn**^**2+**^**with****d****-malate as substrate showing the experimentally determined kinetic rate constants at 25 °C.***Pa*D2HGDH, *P. aeruginosa*d-2-hydroxyglutarate dehydrogenase.

Evidence to support a reversible hydride transfer reaction (Fig. 10) comes from the observed nonzero *y*-intercept in the hyperbolic dependence of the observed rate of flavin reduction as a function of d-malate concentration (Figs. 2 and 4). The ratio of the forward and reverse rate constants for hydride transfer was ∼15 for E-Zn^2+^ with d-malate as a substrate, consistent with the 92% reduction of the enzyme-bound flavin observed when E-Zn^2+^ was mixed anaerobically with d-malate (21). When Zn^2+^ was replaced with Co^2+^ in the enzyme, a ratio of ∼10 was determined from the forward and reverse rate constants for hydride transfer with d-malate as a substrate, suggesting similar mechanistic roles for Zn^2+^ and Co^2+^ in *Pa*D2HGDH. Such reversible flavin reduction has been reported for nonenzymatic metal–flavin complexes (87). *Pa*D2HGDH is the first example to our knowledge of a metallo flavoprotein for which the reversibility of the hydride transfer reaction has been reported.

### Hydride transfer and product release are partially rate limiting for the overall turnover of *Pa*D2HGDH with d-malate and D2HG, irrespective of the bound metal

Evidence to support this conclusion comes from the steady-state kinetics (Tables 1 and 2), rapid-reaction kinetics (Tables 3 and 4), and the kinetic solvent viscosity effects on the steady-state kinetic parameters of the enzyme (Fig. 5 and Table 5). The linear pattern of the viscosity plots of the normalized *k*_cat_ values as a function of the relative solvent viscosity yielded straight lines with positive slopes for all substrates and viscosigens tested, consistent with diffusional processes, such as product release, contributing to the overall turnover of the enzyme (76). Indeed, for a reaction in which the overall turnover is solely limited by hydride transfer, that is, *k*_cat_ = *k*_red_, increasing solvent viscosity would have no effect on the *k*_cat_ value, yielding a slope of 0 in the viscosity plots for the *k*_cat_ parameter (76). For E-Zn^2+^ with d-malate, using Equation A, a rate constant for oxaloacetate release (*k*_P-rel_ in Fig. 10) of 80 ± 30 s^−1^ was estimated from the averaged slope of 0.46 and the experimentally determined *k*_red_ and *k*_rev_ values. A *k*_P-rel_ value of 230 ± 100^2^ s^−1^ was independently estimated from the experimentally determined *k*_red_, *k*_rev,_ and *k*_cat_ values, using Equation B. A rate constant for product release between 80 and 230 s^−1^ is 1.5 to 4.0 times faster than the rate constant for hydride transfer, consistent with both kinetic steps contributing to the overall turnover of the enzyme (88). Similar results were obtained when Zn^2+^ was replaced with Co^2+^, and d-malate with D2HG, consistent with hydride transfer and product release being partially rate limiting for *Pa*D2HGDH turnover irrespective of the substrate and bound metal (88). The tighter binding of D2HG as compared with d-malate suggests a similar binding affinity of the two keto products of the reaction, that is, 2-ketoglutarate and oxaloacetate, which is likely reflected in the charge transfer complex observed with D2HG, but not d-malate, at the end of the reductive half-reaction (18).

(A) $$Slope\;\left(m\right)=\frac{k_{red}+k_{rev}}{k_{red}+k_{rev}+k_{P-rel}}$$

(B) $$k_{cat}=\frac{k_{red}k_{P-rel}}{k_{red}+k_{rev}+k_{P-rel}}$$

### Zn^2+^ activates water in the active site of *Pa*D2HGDH

Evidence supporting this conclusion comes from the pL profile of the *k*_cat_/*K*_*m*_ values for E-Zn^2+^ with d-malate as substrate in H_2_O or D_2_O (Fig. 6), showing a positively sloped limb at pL values below ∼8.0. In the absence of substrate, Zn^2+^ mediates the ionization of an active-site water molecule to yield a hydroxide ion with a p*K*_a_ value between 7.0 and 8.0. This pattern is similar to other Zn^2+^-dependent metalloenzymes that require a Zn^2+^-bound OH^−^ with p*K*_a_ values between 7.0 and 9.0 for catalysis, such as, for example, carbonic anhydrase, alcohol dehydrogenase, carboxypeptidase A, and thermolysin (61, 64, 66, 78, 79, 80, 89, 90, 91, 92, 93, 94), for which a positively sloped limb in pL profiles was observed. Independent evidence for Zn^2+^ mediating the activation of water to yield a hydroxide ion comes from the increase in the flavin N3 atom p*K*a value from 10.7 to 11.9 when Zn^2+^ is bound in the enzyme’s active site, as reported in the accompanying article (21). Such an increase in the flavin N3 atom p*K*a value stems from the proximity of the Zn^2+^ hydroxide to the enzyme-bound flavin reducing the likelihood of flavin deprotonation. A Zn^2+^-hydrate was recently shown in the crystal structure of the human enzyme devoid of a substrate, providing structural evidence for the presence of water in the active site of the resting enzyme (77).

In conclusion, this study highlights the role of Zn^2+^ in substrate orientation and activation during *Pa*D2HGDH catalysis and proposes a catalytic mechanism for the enzyme (Fig. 7). The importance of this study stems from the need to elucidate the catalytic mechanism of *Pa*D2HGDH for the informed design and development of targeted therapeutics against *P. aeruginosa* infections. The study establishes that the enzyme’s active site metal binds to and orients the hydroxy acid substrate through a bidentate interaction, the metal activates the substrate to yield an alkoxide in a process that initiates hydride transfer to the flavin, and flavin reduction is reversible and partially rate limiting for the overall turnover of the enzyme. This study is the first to propose a catalytic mechanism for *Pa*D2HGDH and provides insight into the roles of Zn^2+^ during catalysis. Given that the enzyme is a newly identified metallo flavoprotein, an understanding of the catalytic mechanism and the identification of the important groups involved in catalysis will provide insights into the mode of action of D2HGDHs and allow for a better understanding of the catalytic mechanisms of metallo flavoproteins.

## Experimental procedures

### Materials

The *Pa*D2HGDH pET20b(+) plasmid harboring the PA0317 gene was designed in-lab and purchased from GenScript. The plasmid was sequenced to verify the presence of the wildtype gene. *Escherichia coli* strain Rosetta(DE3)pLysS was from Novagen. Bovine serum albumin was purchased from Promega. Luria–Bertani agar, Luria–Bertani (LB) broth, chloramphenicol, IPTG, lysozyme, PMS, and PMSF were obtained from Sigma–Aldrich. Ampicillin was purchased from ICN Biomedicals. d-2-hydroxyglutarate was purchased from MilliporeSigma. D-malate was purchased from Alfa Aesar. Deuterium oxide, deuterium chloride, and sodium deuteroxide were purchased from Cambridge Isotope Laboratories, Inc. Glucose, glycerol, and all the other reagents were of the highest purity commercially available.

### Expression and purification of *Pa*D2HGDH

To obtain pure enzyme for kinetic studies, a 10 ml LB broth medium containing 100 μg/ml ampicillin and 34 μg/ml chloramphenicol was inoculated with frozen stocks of *E. coli* cells Rosetta(DE3)pLysS harboring the *Pa*D2HGDH pET 20b(+) plasmid. The cell cultures were used to inoculate 1 l LB broth and incubated on a rotatory plate at 37 °C and 180 rpm for 18 h. Protein expression was induced with 100 μM IPTG when cell density reached an absorbance of ∼0.6 at 600 nm. The temperature of the culture was then lowered to 18 °C while shaking on a rotatory plate at 180 rpm. After 17 h of expression, the cells were harvested by centrifugation for 30 min at 2800*g* and 4 °C.

To obtain the Zn^2+^-bound enzyme, the lysis buffer containing 1 mM PMSF, 2 μg/ml DNase or RNase, 4 mg/ml lysozyme, 5 mM MgCl_2_, 300 mM NaCl, 10 mM imidazole, 10% glycerol, 1 mM ZnCl_2_, and 20 mM NaPO_4_, pH 7.4, was used to resuspend the wet cell paste in a ratio of 1 g of the wet cell paste to 4 ml of lysis buffer. The suspended cells were then incubated for 30 min on ice while stirring. The resulting slurry was sonicated in five cycles of 5 min each with 5 min off intervals, and then the cell debris was removed by centrifugation at 11,200*g* for 30 min. The supernatant (cell-free extract) was purified to homogeneity using a nickel–nitrilotriacetic acid column, equilibrated with buffer A (20 mM NaPO_4_, 10 mM imidazole, 300 mM NaCl, 1 mM ZnCl_2_, and 10% glycerol, pH 7.4). The purification was carried out using a Unicorn ÄKTA Start purification system. Elution of the bound protein was through a gradient from 0 to 100% buffer B (20 mM NaPO_4_, 500 mM imidazole, 300 mM NaCl, 1 mM ZnCl_2_, and 10% glycerol, pH 7.4), with *Pa*D2HGDH eluting at ∼40% buffer B. The solution containing the purified protein, typically 15 ml, was dialyzed against five 2 l changes of 10% glycerol, 20 mM NaPO_4_, 1 mM ZnCl_2_, pH 7.4, for 24 h, at 4 °C. The purified enzyme (E-Zn^2+^) was stored in single-use aliquots in 10% glycerol, 25 mM NaPO_4_, pH 7.4, at −20 °C and was stable for at least 6 months.

To obtain the enzyme loaded with Co^2+^, the aforementioned protocol was followed. However, all reaction buffers contained 1 mM CoCl_2_ instead of 1 mM ZnCl_2_, to yield the E-Co^2+^ enzyme.

### Reductive-half reaction

To determine the *K*_*d*_ values for D2HG and d-malate with the recombinantly expressed *Pa*D2HGDH purified in the presence of Zn^2+^ (E-Zn^2+^), the reduction of the enzyme-bound flavin was followed by monitoring the decrease in absorbance at 450 nm upon mixing E-Zn^2+^ with varying concentrations of the reducing substrate. The time-resolved absorbance spectroscopy of the reduction of E-Zn^2+^ with D2HG or d-malate was carried out with an SF-61DX2 Hi-Tech KinetAsyst high-performance stopped-flow spectrophotometer equipped with a photomultiplier detector and thermostated with a water bath at 25 °C under anaerobic conditions. The reductive half-reaction was performed under pseudo–first-order conditions where the enzyme concentration after mixing with substrate was ∼9 μM and that of the reducing substrate was between 80 and 800 μM (D2HG) or 0.6 and 60 mM (d-malate). The enzyme was equilibrated with 25 mM NaPO_4_, pH 7.4, using a PD10 column. Equal volumes of the enzyme and the reducing substrate were mixed in the stopped-flow spectrophotometer in single-mixing mode following established procedures with an instrument dead time of 2.2 ms.

To determine the *K*_*d*_ values for D2HG and d-malate with the recombinantly expressed *Pa*D2HGDH purified in the presence of Co^2+^ (E-Co^2+^), the aforementioned protocol was repeated using E-Co^2+^ in place of E-Zn^2+^.

The stopped-flow traces were fit to Equation 1 using the KinetAsyst 3 (TgK-Scientific) software. The equation describes a double-exponential process in which *A* represents the absorbance at 450 nm at time *t*, *B*_*1*_ and *B*_*2*_ represent the amplitudes of the decrease in absorbance, *k*_obs1_ and *k*_obs2_ represent the observed rate constants for the change in absorbance associated with flavin reduction. *C* is an offset value accounting for the nonzero absorbance of the enzyme-bound reduced flavin at infinite time.

(1) $$A={B_{1}}^{{-k}_{obs1}t}+{B_{2}}^{{-k}_{obs2}t}+C$$

The concentration dependence of the observed rate constants for flavin reduction was analyzed with Equation 2, which describes a hyperbolic trend. In this equation, *S* represents the concentration of the organic substrate, *k*_red_ is the rate constant for flavin reduction at saturating substrate concentrations, *k*_rev_ is the reverse rate of enzyme catalysis that reports on the conversion of the enzyme–product complex to the enzyme–substrate complex, and *K*_*d*_ is the apparent dissociation constant for substrate binding.

(2) $$k_{obs}=\frac{k_{red}S}{K_{d}+S}+k_{rev}$$

### Enzyme activity and steady-state kinetics

To investigate the effects of metal introduction on the kinetic properties of E-Zn^2+^ and E-Co^2+^, the enzymes were analyzed under steady-state conditions by monitoring the initial rates of oxygen consumption with a computer-interfaced Oxy-32 oxygen-monitoring system (Hansatech Instruments Ltd) thermostated with a water bath. The steady-state kinetic parameters of E-Zn^2+^ or E-Co^2+^ were determined by varying the concentrations of the reducing substrate D2HG (0.025–0.4 mM) or d-malate (1.6–40 mM) and the artificial electron acceptor PMS (0.02–0.5 mM) in 25 mM NaPO_4_, pH 7.4, and 25 °C with ∼7 nM enzyme.

Data analysis was conducted using the KaleidaGraph software (Synergy Software) and Enzfitter software (Biosoft). To determine the steady-state kinetic mechanism of E-Zn^2+^, the best fit of the initial rates of enzyme reaction was obtained using the equation that describes a ping–pong bi–bi steady-state process, consistent with the data obtained previously for *Pa*D2HGDH purified without metals (18).

### Kinetic solvent viscosity effects

To investigate the effects of solvent viscosity on the kinetic properties of *Pa*D2HGDH, the steady-state kinetic parameters were determined by varying the concentration of D2HG (0.025–0.4 mM) or d-malate (1.6–40 mM), with a reaction buffer of 25 mM NaPO_4_, pH 7.4, and 25 °C, containing varying amounts of glycerol (0–40%, m/m, η_rel_ = 1.0–3.5 cP). Assay reaction mixtures were equilibrated at atmospheric oxygen for at least 2 min before the reaction started with the addition of the enzyme. The experiment was repeated using glucose as a viscogen (0–34%, m/m, η_rel_ = 1.0–3.6 cP) for both substrates. The relative viscosities of all viscogens were comparable. The data from the viscosity effects on the *k*_cat_ and *k*_cat_/*K*_*m*_ values for both substrates were fit to Equation 3, where (*Kinetic Parameter*)_o_ and (*Kinetic Parameter*)_η_ are the values for the kinetic parameters in the absence and presence of viscogen, respectively. *S* is the degree of viscosity dependence, and *η*_*rel*_ is the relative viscosity of the buffered solution.

(3) $$\frac{{\left(Kinetic\;Parameter\right)}_{o}}{{\left(Kinetic\;Parameter\right)}_{\eta }}=S\;\left(\eta_{rel}-1\right)+1$$

### pL profiles of E-Zn^2+^

To determine the effect of pH on the kinetic parameters of E-Zn^2+^, a series of enzymatic apparent steady-state kinetic assays were carried out over a pH range of 6.0 to 9.0 at varying concentrations of d-malate as the reducing substrate and fixed 2 mM saturating concentration of PMS as an artificial electron acceptor. The assays were carried out in air-saturated 25 mM Goods buffer (0.1 M ACES, 0.052 M Tris, and 0.052 M ethanolamine) at 25 °C with d-malate concentrations ranging from 1.6 to 40 mM. The final enzyme concentration was ∼7 nM in 1 ml reaction volumes. Enzyme activity was investigated by monitoring the initial rates of oxygen consumption with a computer-interfaced Oxy-32 oxygen-monitoring system (Hansatech Instruments Ltd) thermostated with a water bath. The enzyme was stable at all pL values tested.

For the determination of the solvent kinetic isotope effects, buffers and substrate solutions were prepared using 99.9% D_2_O by adjusting the pD value with NaOD. The pD values were determined by adding 0.4 to the pH electrode readings (95). For all steady-state kinetic isotope effects, activity assays were carried out as described previously and D_2_O was used in place of H_2_O. For each pH or pD (pL) value tested, the reaction was carried out by alternating between the protonated and deuterated reaction mixtures.

The pL dependence of the *k*_cat_ and *k*_cat_/*K*_*m*_ values was determined by fitting the data with Equation 4, which describes a bell-shaped curve with a slope of +1 on the increasing limb at low pL, a curved region with a limiting pL-independent value, that is, C, and a slope of −1 on the decreasing limb at high pL. The p*K* values for the unprotonated and protonated groups are denoted by p*K*_a1_ and p*K*_a2_, respectively. *Y* is the kinetic parameter under investigation, that is, *k*_cat_ or *k*_cat_/*K*_*m*_.

(4) $$\log\;Y=\log\;\frac{C}{1+\frac{10^{-pL}}{10^{{-pK}_{a1}}}+\frac{10^{{-pK}_{a2}}}{10^{-pL}}}$$

## Data availability

All data are contained within the article.

## Conflict of interest

The authors declare that they have no conflicts of interest with the contents of this article.

## Footnotes

1 Conventionally, solvent kinetic isotope effects coupled with pL profiles are used to demonstrate the catalytic roles of metal ions in metalloenzymes (96, 97). The presence of an activated water hydroxide that gains a proton during rate-limiting kinetic steps in catalysis would be established by an observed inverse solvent kinetic isotope effect (24, 28, 29, 30, 31, 32, 33, 34, 35, 96, 97). On the other hand, systems that do not contain metals, such as flavoenzymes, employ active site bases like histidine residues and yield normal solvent kinetic isotope effects when protons are in flight in the rate-limiting kinetic steps (81, 82, 97). In this study, however, because of the p*K*a values for the protonated and unprotonated groups being overlapped, the pL effects on the *k*_cat_ values did not provide a pL-independent region that would allow for the determination of either a normal or an inverse solvent kinetic isotope effect. Hence, solvent kinetic isotope effects data could not be used to draw mechanistic conclusions for *Pa*D2HGDH.

2 Despite the large propagated errors associated with the two independent calculations, the two estimated *k*_P-rel_ values are comparable to the rate constant for hydride transfer, allowing for the conclusion that both hydride transfer and product release contribute to the overall enzyme turnover to be drawn.
